# Reverse total shoulder arthroplasty in patients with type B2, B3, and type C glenoids: comparable clinical outcome to patients without compromised glenoid bone stock—a matched pair analysis

**DOI:** 10.1007/s00402-021-03939-4

**Published:** 2021-06-02

**Authors:** Rafael Loucas, Philipp Kriechling, Marios Loucas, Rany El Nashar, Christian Gerber, Karl Wieser

**Affiliations:** Department of Orthopedics, Balgrist University Hospital, University of Zurich, Forchstrasse 340, 8008 Zurich, Switzerland

**Keywords:** Glenoid Dysplasia, Retroversion, Reverse total shoulder arthroplasty, Osteoarthritis of the Shoulder, Shoulder arthroplasty

## Abstract

**Background:**

Primarily posterior bone deficient (dysplastic) (Walch type C) or secondarily eroded (Walch type B2 or B3) glenoids represent a surgical challenge for shoulder arthroplasty. Due to the posteriorly static decentered head, reverse total shoulder arthroplasty (RTSA) is often considered as the treatment of choice. The purpose of this study is to report the clinical and radiographic outcomes, complications and reoperations of RTSA for posteriorly deficient glenoids.

**Materials and methods:**

All patients who underwent RTSA for osteoarthritis secondary to underlying glenoid deficiency (Walch type B2, B3 and C) between 2005 and 2018 (study group), were identified from our institutional shoulder arthroplasty database and gender- and age-matched to a cohort of patients with normal glenoid bone stock (control group). Longitudinal pre- and postoperative clinical [Constant–Murley (CS) score, Subjective Shoulder Value (SSV)] and radiographic outcomes were assessed.

**Results:**

We included 188 patients (94 in each group). The median follow-up was 43 ± 26 (24–144) months in the study group and 59 ± 32 (24–124) months in the control group. The glenoid deficiency was addressed by using glenoid bone reconstruction. The surgical site complication and revision rate of RTSA in patients with bony deficient glenoids were 17% and 7%. Although glenoid loosening was slightly higher in the study group (5 vs. 2), overall no significant differences were found between the study and control groups in satisfaction scores, preoperative and postoperative absolute and relative Constant scores, complication and revision rates, respectively.

**Conclusion:**

Reverse total shoulder arthroplasty (RTSA) seems to be a valuable treatment option for patients with primary (dysplasia) or secondary (wear) posterior glenoid deficiency. Although severe glenoid bone loss seems to be a risk factor for glenoid component failure, the overall complication and revision rates as well as clinical and radiographic outcome are comparable to RTSA in patients without compromised glenoid bone stock.

**Level of evidence:**

Level III: case–control study

## Introduction

Posterior glenoid deficiency can be either related to a developmental anomaly of the scapula, better known as glenoid dysplasia (GD), or a progressive process with increasing posterior glenoid wear. Both entities are still not fully understood but, at least to a certain degree associated with a static posterior subluxation of the humeral head, which further increases posterior glenoid cartilage and bone wear and therefore leads to eccentric osteoarthritis of the glenohumeral joint. Glenoid dys- or hypoplasia may occur as a primary isolated condition or in association with various syndromes [1]. Once thought to be a rare condition, more recent studies have shown that the incidence of glenoid hypoplasia ranges from 18 to 35% [2]. The glenoid morphology was originally classified by Walch in 1999 and has recently been modified [3, 4].

Furthermore, Denard [5] and Walch et al. [3] used CT scans to classify morphologic features of the glenoid in primary glenohumeral osteoarthritis (GHOA) based on the glenoid version and the glenohumeral subluxation index. The classification provides an anatomic descriptive characterization of primary glenohumeral osteoarthritis. According to Walch, 24% of glenoids in GHOA shows a biconcave secondary posterior erosion (type B2) or excessive posterior retroversion greater than 25° and are dysplastic in origin (type C) [3, 6, 7]. Recent studies provide evidence that biconcave posteriorly eroded B2 glenoids can progress over time leading to severe posterior bone erosion and secondarily increased glenoid retroversion, mimicking primary dysplasia. Such type B3 glenoids according to the modified Walch classification [8, 9], are defined as a monoconcave glenoids with posterior bony wear and severe pathologic retroversion of at least 15° or at least 70% posterior humeral head subluxation, or both.

In the case of symptomatic secondary GHOA joint replacement might become necessary if conservative measures are exhausted. However, such posteriorly deficient glenoids still represent an intellectual and surgical challenge and are technically demanding even for experienced shoulder surgeons. Anatomic total shoulder arthroplasty, with eccentric reaming, posterior glenoid bone grafting, or posterior augmented glenoid components are associated with a high failure rate mainly due to early glenoid loosening or dislocation [3, 10–15]. Therefore, semi-constrained reverse total shoulder arthroplasty (RTSA) has come into favor in recent years but its outcome seems also to be affected by such challenging glenoids [6, 16].

The purpose of this study was to report the clinical and radiographic outcomes, complications and reoperations of RTSA in a large series of patients with posterior glenoid deficiency (Walch type B2, B3 and C), and compare these results in a matched pair analysis to a cohort of patients with primary RTSA and normal glenoid bone stock.

## Materials and methods

This was a retrospective matched case–control study of the clinical and radiologic outcomes of RTSA for patients with glenoid dysplasia type B2, B3, and C.

Between 2005 and 2018, 120 primary RTSAs for osteoarthritis secondary to underlying posterior glenoid insufficiency (Walch type B2, B3 and C) [8] with and without rotator cuff deficiency were performed in our institution. This study was approved by the ethics committee of the University of Zurich (ID 2018-01494) and conducted following the Helsinki Declaration.

### Patients selection

Our institutional RTSA database documents 823 consecutive primary RTSA procedures between January 2005 till March 2018. Of these, 120 surgeries were performed in patients with various degrees of posterior glenoid deficiency (type B2, B3 and C according to the modified Walch classification [8]).

To be included in the study, patients had to have posterior glenoid insufficiency (Walch type B2, B3 and C), the operation had to be a primary RTSA, and a complete clinical and radiographic follow-up as well as informed consent to participate in the study had to be available.

If, in addition to primary RTSA, other surgical measures (e.g. latissimus dorsi transfer) or revision arthroplasties were performed, the patient was excluded.

This study group was gender and age matched to a cohort of patients with primary RTSA and normal glenoid bone stock.

**Fig. 1:**
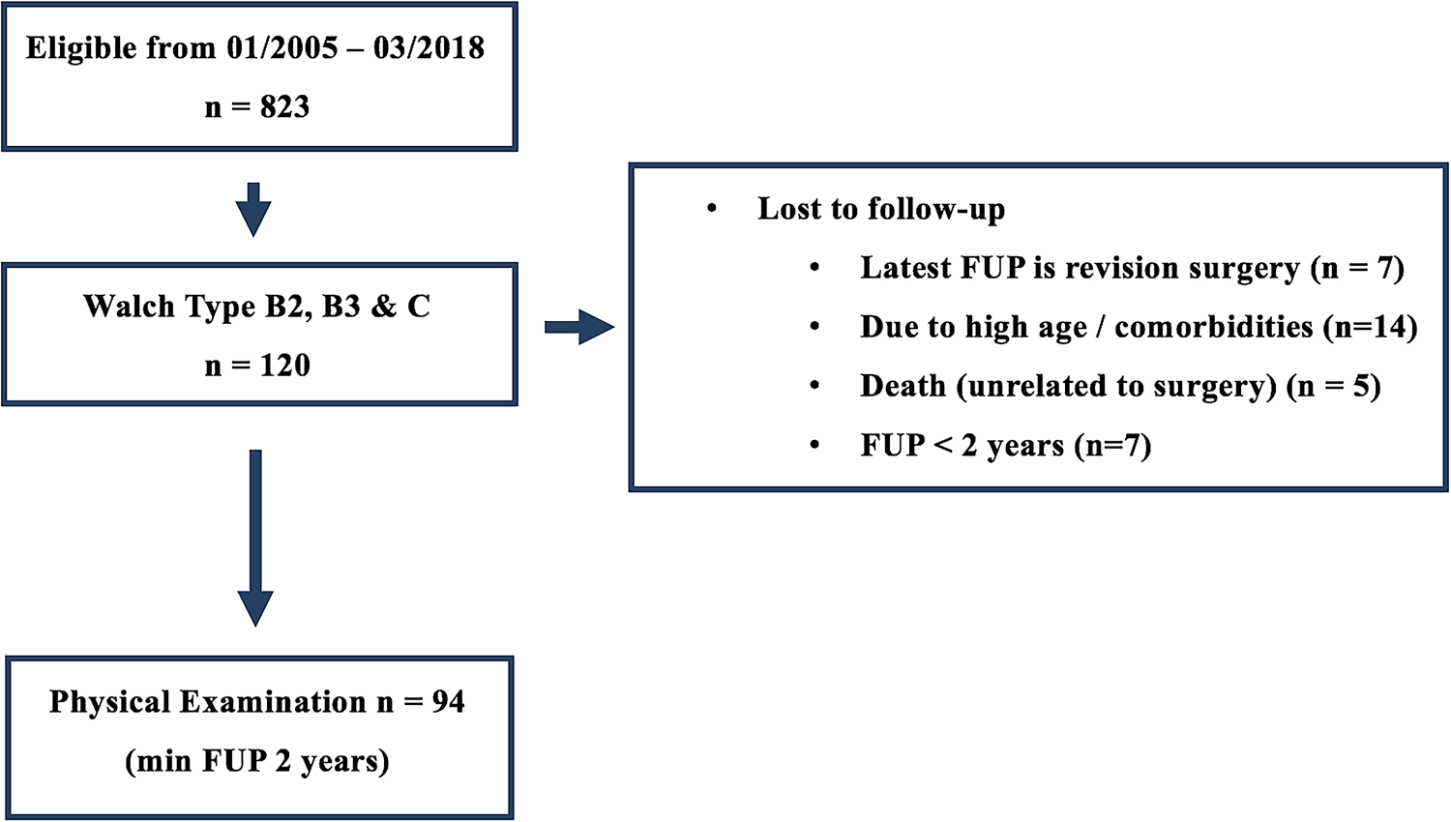
Flow-chart demonstrating patient selection. *FUP* follow-up period, *ORIF* open reduction and internal fixation

Of the identified 120 shoulders (120 patients), 7 patients (6%) were revised. Three (2%) of them because of superior glenoid dislocation (8, 15 and 36 months), two (2%) because of greater tuberosity displacement (1 and 2 months), one (1%) for a posttraumatic humeral fracture (24 months), one (1%) for an acromion fracture (3 months). Additionally, 14 patients (11.6%) were unable to travel for further examination because of high age or poor health status and 5 (4%) patients had passed away before regular follow-up, all unrelated to the surgical procedure. These twenty-six patients (study group) were included in the failure analysis but had to be excluded from further clinical and radiological analysis (Fig. 1).

### Clinical and radiographic assessment

Clinical and radiographic examinations were performed preoperatively and approximately 1 year, 2–5 years, 5–8 years, 8–10 years, and more than 10 years postoperatively. All patients underwent a standardized clinical and conventional radiographic examination by an examiner different from the operating surgeon sequentially at each regular consultation. The clinical examination included measurement of active and passive ranges of motion using a handheld goniometer and assessment of the absolute (aCS) and relative Constant scores (rCS) [17, 18], and Subjective Shoulder Value (SSV) [19]. Patients rated their overall postoperative satisfaction as excellent, good, fair, or poor. Abduction strength in the scapular plane was measured with a validated electronic dynamometer (Isobex; Cursor, Bern, Switzerland) [20]. Preoperatively and postoperatively, standardized true anteroposterior, axillary lateral, and scapular lateral (Neer view) radiographs were obtained for all patients. On preoperative anteroposterior radiographs, grades of rotator cuff arthropathy were identified according to the Hamada classification [21]. On preoperative computed tomography scans, glenoid bone stock and form were graded according to the Walch [3] (mid axial cut) and the Favard classification [22]. Two different and blinded readers (RL and RE) independently evaluated each CT-scan.

Outcome measurements evaluated on the postoperative radiographs were inferior scapular notching according to the Sirveaux classification [23], radiolucency, heterotopic ossification, glenoid or humeral loosening, and glenoid or acromial fracture.

### Surgical technique

All procedures were performed with the patient in a beach chair position, through a deltopectoral approach, by a specialized shoulder surgeon. The operations were done in a standardized manner: antibiotic prophylaxis with Cefuroxim 1.5 g (Fresenius Kabi, Switzerland) was administered intravenously 30 min before skin incision. General anesthesia in combination with an interscalene block was used in 83 cases and regional anesthesia and sedation alone in 37 cases. If the subscapularis tendon was intact, it was elevated off the lesser tuberosity and reattached before wound closure. The subscapularis tendon was repaired in 61 shoulders (65%) in the study group and 63 shoulders (67%) in the control group. If a type B2, B3 or C glenoid was present, depending on the intraoperative site, the surgeon decided whether to perform autografting with the humeral head to correct the retroversion (23 cases) or iliac crest autograft (1 case) or use of osseous allograft (2 cases).

The Anatomical ShoulderTM Inverse/Reverse SystemTM (Zimmer) was implanted in 93 shoulders (99%) and in one shoulder (1%) of all cases Delta III (DePuy Synthes) were used as implants on the humeral side. In the majority of procedures (*n* = 64; 68%) a standard size glenoid baseplate was used: (Anatomical ShoulderTM System glenoid component (Zimmer Biomet, Warsaw, Indiana, USA) (*n* = 51; 54%); Trabecular metal 15 mm peg (Zimmer Biomet, Warsaw, Indiana, USA) (*n* = 13; 14%). In 32% (*n* = 30), a long-pegged glenoid base plate was used: (Aequalis Reversed II (Tornier, Amsterdam, The Netherlands) (*n* = 8; 9%); Trabecular metal Long peg (25 or 30 mm) (Zimmer Biomet, Warsaw, Indiana, USA) (*n *= 21; 22%), Delta III (DePuy International, Leeds, UK) (*n* = 1; 1%). In the control group, in all shoulders (100%), the ShoulderTM Inverse/Reverse SystemTM (Zimmer) was used on the humeral side. In the majority of procedures (*n* = 92; 98%) a standard size glenoid baseplate was used: (Anatomical ShoulderTM System glenoid component (Zimmer Biomet, Warsaw, Indiana, USA) (*n* = 80; 85%); Trabecular metal 15 mm peg (Zimmer Biomet, Warsaw, Indiana, USA) (*n* = 12; 13%). In 2% (*n* = 2), a long-pegged glenoid base plate was used: (Aequalis Reversed II (Tornier, Amsterdam, The Netherlands) (*n* = 1; 1%) and Delta III (DePuy International, Leeds, UK) 1% (*n* = 1; 1%).

**Fig. 2:**
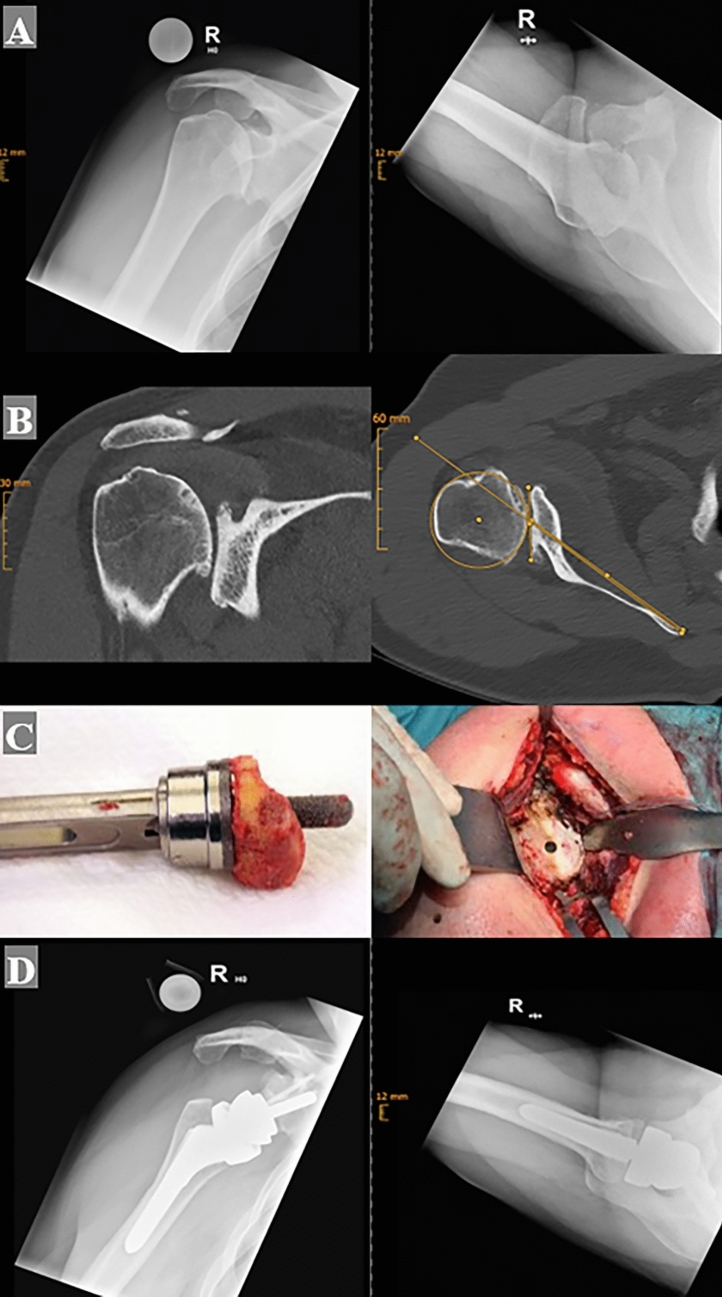
Fifty-seven-year-old man with primary glenohumeral osteoarthritis and a posterior bony deficiency (Walch type C). Preoperative radiograph and computed tomography–scan (**a** and **b**), intraoperative reconstruction of the glenoid using of a structural humeral head autograft (**c**). Post-operative radiograph (**d**)

All patients received a preoperative CT scan, where the glenoid version and bone stock were assessed. According to this CT, the position of the glenoid baseplate was planned in neutral version and neutral to slight inferior tilt. We aimed for at least 70% surface seating of the baseplate. If this could not be achieved posterior (-superior) glenoidal bone grafting was performed without excessive lateralization of the glenoid component (Fig. 2). This was necessary in 26 cases (28%) of the final study group with posterior deficient glenoid bone stock (and overall in 35 cases of the primary available 120 patients). Bone graft was harvested in 23 cases from the humeral head in 1 case from the iliac crest and in 2 cases osseous allograft was used.

### Data collection, statistical analyses and literature review

Matching was performed in a 1:1 pattern of cases to controls for age (± 5 years), and sex. Data were mainly non–normally distributed, and medians, standard deviation (SD), and odds ratio (OD) are provided. The patient’s data were collected in REDCap® Electronic Data Capture system version 8.6.1 (Vanderbilt University, 1211 Medical Center, TN 37232, USA) anonymously [24].

The normal distribution of variables was tested with the Shapiro–Wilk-test and compared pre- and postoperative scores with the paired *t* test (parametric data) or the Wilcoxon-ranksum-test (non-parametric distribution). Fisher's exact test was used for categorical variables. The alpha level was set at 0.05. All the statistical analyses were conducted using the SPSS software v24.0 (IBM, New York, USA).

The interobserver reliability of the assessments of the Walch classification of the posterior glenoid insufficiency (type B2, B3 and C type) was measured by calculating the intraclass correlation coefficient (ICC) for absolute agreement, with 1 indicating perfect reliability.

## Results

### Patients and demographics

Ninty-four shoulders (94 patients) with bony deficiency were treated with RTSA and were available for analysis (study group). The underlying pathology was osteoarthritis in 40 cases (43%), massive rotator cuff tears in 25 cases (26%), conversion from fracture treatment with ORIF in 16 cases (17%), avascular necrosis (AVN) of the humeral head in 8 cases (8.5%), primary fracture treatment in 4 cases (4%), and of crystalline arthropathy in one case (1%).

36 patients had a Walch B2 deformity, 28 patients had a Walch B3 glenoid, and 30 patients had a Walch C glenoid (inter-rater agreement 0.93). The mean glenoid retroversion according to the Friedmann method [25] was 19.5 ± 10.3°.

These patients were matched with 94 shoulders in 94 patients without posterior glenoid deficiency, treated with RTSA (control group). The underlying pathology in this group was a massive rotator cuff tear in 70 cases (76%), osteoarthritis in 7 cases (8%), primary fracture treatment in 7 cases (8%), instability arthropathy in five cases (5%), and avascular necrosis (AVN) of the humeral head in 3 cases (3.3%). All patients (*n* = 94) had a Walch A1 glenoid. The mean glenoid version angle of all shoulders according to the Friedmann method [25], was 2.4 ± 5°. The median follow-up was 43 ± 26 (24–144) months in the study group and 59 ± 32 (24–124) months in the control group. Detailed demographic data (age, sex, BMI, follow-up time, ASA, weight, height) are presented in Table 1.

**Table 1:** Demographic data for the study groups

	Study group Mean ± STD (min, max)	Control group Mean ± STD (min, max)	*p* value	Test
Age at surgery (years)	70 ± 10 (44; 87), *n* = 94	71 ± 10 (43; 88), *n* = 94	0.721	MWU
Follow-up time (months)	43 ± 26 (24; 144), *n* = 94	59 ± 32 (24; 124), *n* = 94	0.001	MWU
Gender	53 female	58 female	0.553	Fisher
	41 male	36 male		
Side	59 right	57 right	0.881	Fisher
	35 left	37 left		
ASA			0.179	MWU
I	0	0	–	–
II	6	5	–	–
III	59	70	–	–
IV	29	19	–	–
V	0	0	–	–
Weight	76 ± 17 (46; 159), *n* = 94	74 ± 17 (47; 135), *n* = 94	0.512	MWU
Height	167 ± 10 (139; 196), *n* = 94	165 ± 10 (134; 186), *n* = 94	0.151	MWU
Body Mass Index	27 ± 6 (17; 63), *n* = 94	27 ± 6 (18; 50), *n* = 94	0.969	MWU

Values are given as mean ± SD [95% confidence interval] or (minimum; maximum)

### Clinical outcome

**Fig. 3:**
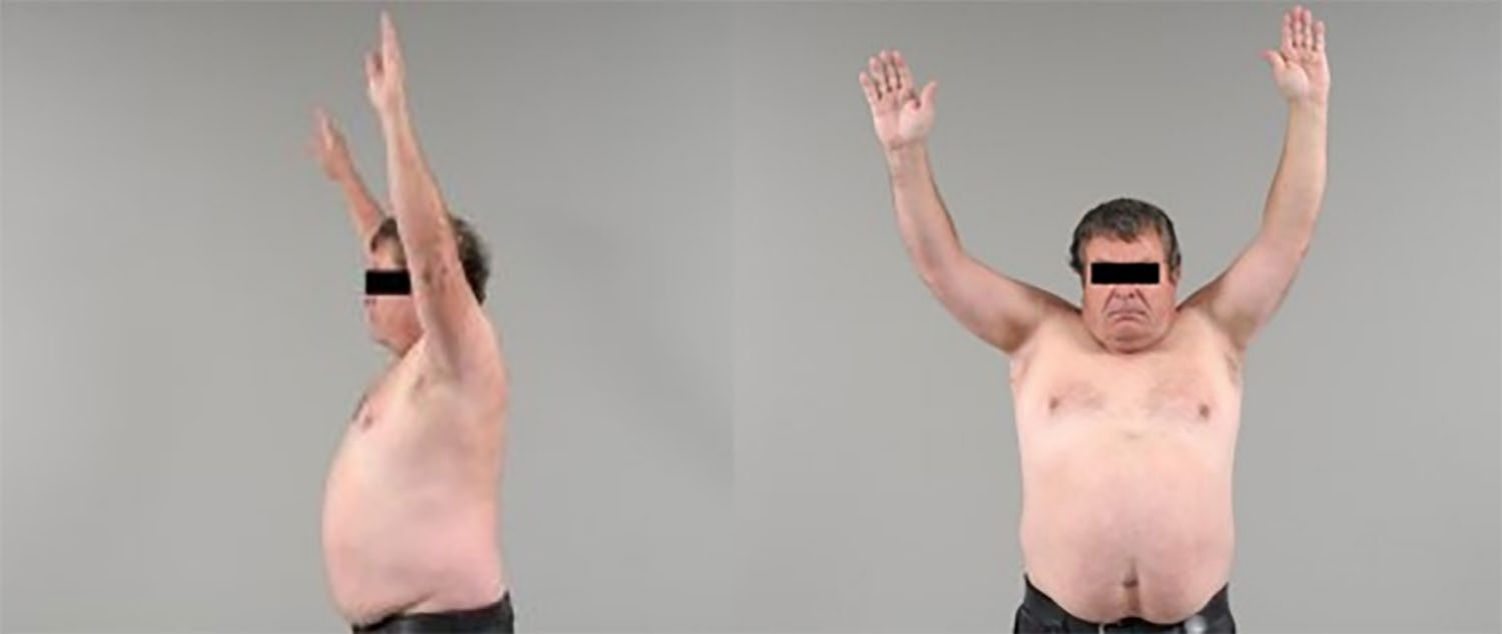
Range of motion at two months postoperatively

RTSA in patients with bony deficient glenoids (study group) resulted in considerable improvement of absolute and relative shoulder scores (absolute CS from 36 to 67 points, relative CS from 45 to 80%), pain (CS pain score from 6 to 14 points), overhead function (flexion from 89 to 119°), force (1.7–3.4 kg) and patient satisfaction (SSV from 36 to 80%). No significant differences were found between the study and control groups in preoperative and postoperative absolute and relative Constant scores, patients' satisfaction, or any postoperative CS sub-values. (Table 2 and Fig. 3).

**Table 2:** Diagnosis and pre- and postoperative scores of the 188 patients available for personal follow-up

	Study group	Control group	
Constant absolute preop	36 ± 16 (8; 70), *n* = 91	36 ± 16 (4; 70), *n* = 94	0.969
Constant absolute postop	67 ± 17 (13; 92), *n* = 94	65 ± 17 (15; 87), *n* = 94	0.546
Constant relative preop	45 ± 18 (10; 84), *n* = 91	45 ± 19 (4; 83), *n* = 94	0.846
Constant relative postop	80 ± 20 (13; 106), *n* = 94	79 ± 20 (17; 102), *n* = 94	0.635
SSV preop	36 ± 18 (0; 80), *n* = 88	34 ± 20 (0; 100), *n* = 91	0.548
SSV postop	80 ± 23 (10; 100), *n* = 92	77 ± 22 (20; 100), *n* = 92	0.397
Flexion preop	89 ± 31 (10; 170), *n* = 91	89 ± 47 (0; 170), *n* = 94	0.967
Flexion postop	119 ± 29 (0; 165), *n* = 94	121 ± 29 (20; 160), *n* = 94	0.784
Abduction preop	75 ± 32 (10; 160), *n* = 91	79 ± 43 (0; 170), *n* = 94	0.586
Abduction postop	129 ± 38 (0; 180), *n* = 94	131 ± 38 (25; 180), *n* = 94	0.780
ER preop	22 ± 23 (− 30; 70), *n* = 91	31 ± 28 (-20; 90), *n* = 94	0.013
ER postop	28 ± 20 (− 40; 65), *n* = 94	29 ± 19 (− 20; 70), *n* = 94	0.639
IR preop	4 ± 2 (0; 8), *n* = 91	5 ± 3 (0; 10), *n* = 94	0.001
IR postop	5 ± 2 (0; 10), *n* = 94	5 ± 3 (0; 10), *n* = 94	0.645
Force preop	1.7 ± 2.3 (0; 10.1), *n* = 91	1.6 ± 4 (0; 34.9), *n* = 94	0.736
Force postop	3.4 ± 2.3 (0; 10.6), *n* = 94	2.9 ± 2 (0; 8.2), *n* = 94	0.116
Pain preop	6 ± 4 (0; 15), *n* = 91	6 ± 4 (0; 15), *n* = 94	0.426
Pain postop	14 ± 3 (4; 15), *n* = 94	13 ± 3 (1; 15), *n* = 94	0.277

Values are given as mean ± SD [95% Confidence Interval] or (minimum; maximum)

### Complications and revisions

We identified 7 (7%) intraoperative and 16 (17%) postoperative complications in the study group compared with 10 (11%) intraoperative and 21 (22%) postoperative complications in the control group. Intraoperative complications were non- to minimally displaced periprosthetic fractures of humeri in 6 (6%) vs. 9 cases (10%) respectively, and intraoperative cement extravasation in 1 case (1%) in each group. All of them healed under conservative treatment without any associated radiological complications (i.e. stem loosening).

Seven of the primary potentially available 120 patients with RTSA in glenoid deficiency (6%) required complication-related reoperations. Three (2%) of them were revised for superior glenoid component dislocation, two (2%) because of displaced fracture of the tuberosity, one (1%) for posttraumatic humeral fracture, and one (1%) for acromion fracture. The remaining complications (10%) were two cases (2%) of glenoid loosening potentially related to a fall. In three (3%) cases a transient neurologic lesion of the radial nerve or the axillar nerve were recorded. Five cases (5%) had periprosthetic fractures. Two cases (2%) of the humeral stem and in three cases (3%) of the acromion. Four patients in the study group and one patient in the control group with glenoid failures showed a severe bony deficiency (3 patients with Walch type B3, one Walch type C and one Walch type B2 glenoid). In 3 of them, intraoperative bony augmentation with a long peg glenoid baseplate was performed.

The overall revision rate in the control group was 4% (four cases). Detailed information about complications and revisions are presented in Table 3.

**Table 3:** Complications and reoperations

Complication	Study group			Control group		
	Intraoperative	Postoperative	Reoperation	Intraoperative	Postoperative	Reoperation
Shaft fracture	6	3	1	9	2	2
Glenoid loosening		5	3		2	1
Fracture of greater tuberosity		2	2			
Radial/Axillary nerve palsy		3			7	
Cement extrusion	1			1		
Fracture of acromion		3	1		5	
Wound infection					2	
Wound healing problem					1	1
Hematoma					2	

### Radiographic outcomes

In addition to the three revised glenoids two patients of the study group showed radiographic signs of glenoid loosening. In the control group, there was one revised glenoid and one additional radiographically loose glenoid (Table 3). Scapular notching was recorded in 41 cases (43.9%) in the study group and 35 cases (37.2%) in the control group (*p* = 0.37). Notching was not correlated to inferior outcome in the Constant-Murley-Score or the Subjective Shoulder Value. An asymptomatic radiolucency around the stem was found in four patients, three in the study and one in the control group. The radiological results are summarized in Table 4.

**Table 4:** Radiographic results

Radiologic item	Study group (*n*, %)	Control group (*n*, %)
Scapular notching rate	41%	35%
Grade I	30, 32%	21, 22%
Grade II	8, 9%	9, 10%
Grade III	2, 2%	4, 4%
Grade IV	1, 1%	1, 1%
Heterotopic ossifications	6, 6%	20, 21%
Radiolucency of humeral stem	3, 3%	1, 1%
Pre OP Glenoid retroversion (mean°, SD)	19.5 ± 10.3°	2.4 ± 5°
Humeral head posterior sublaxation (mean, SD)	67 ± 10.6%	49 ± 4.6%
Walch classification Glenoid type: A1/B2/B3/C	0/36/28/30	94/0/0/0
Favard classification Glenoid type: E1/E2/E3/E4/E5	25/43/5/1/14	71/16/2/0/5

## Discussion

This study shows that RTSA in patients with deficient posterior glenoid bone stock (Walch type B2, B3 and C) resulted in considerable improvement of pain (CS pain score from 6 to 14 points), function (absolute CS from 36 to 67 points), force (1.7–3.4 kg) and patient satisfaction (SSV from 36 to 80%) at a mean of 43 months postoperatively. The surgical site complication and revision rate was 17% and 7%. These results were overall comparable and not different (i.e. not inferior) to a gender- and age-matched cohort of patients after RTSA with sufficient glenoid bone stock.

The treatment of posteriorly deficient glenoids with secondary GHOA is still under debate but truly represents, even for experienced shoulder surgeons, an intellectual (i.e. indication) and surgical challenge. Different treatment options have been suggested for anatomic hemi- or total shoulder arthroplasty. Asymmetric reaming, posterior glenoid bone grafting, or posteriorly augmented glenoid components have been used, but peak stress on the posterior glenoid edge still leads to increased failure rates, mainly due to early glenoid loosening or dislocation [15, 26–28].

Therefore, RTSA with its semi-constrained design and increased glenoid fixation strength has come into favor over the last years with early reports showing promising results.

Alentorn Geli et al. [16] recently evaluated the results of 12 RTSA in patients with GHOA secondary to posterior glenoid dysplasia with 28 months of mean follow-up. They reported better pain relief as well as higher rate of satisfactory results after RTSA compared to hemiarthroplasty or total shoulder arthroplasty results from other studies [10–13].

Also Mizuno et al. [29] published a retrospective review evaluating 27 patients with a mean follow-up of 54 months. They reported excellent results for reverse shoulder replacements on patients with glenoid retroversion and functional rotator cuffs. The authors concluded that RTSA is a viable treatment option to solve both the problem of severe glenoid erosion as well as the severe static posterior glenohumeral instability [29]. In a retrospective, multicenter cohort study of 45 patients with GHOA with B1, B2, B3 and C glenoids who underwent RTSA, Collin et al. [30] found that the CS score improved from 30 to 68 (*p* < 0.001). The postoperative complication rate was 6%, and 4% of these patients needed revision surgery.

To our knowledge, this is the first study with the largest sample size [10, 11, 16] using a propensity-matched analysis comparing clinical and radiographic results after RTSA in patients with posterior glenoid deficiency compared to patients with normal glenoid bone stock. We are, however, aware of some limitations of this investigation. Although the patient's data were prospectively enrolled in our institutional RTSA database, this study does not fulfill all criteria of a prospective design. Even if propensity-score matching may minimize selection bias, this remains a nonrandomized study with the inherent limitation of such study design. Different prosthetic implants had been used over time and decision to posterior glenoid bone grafting was to some degree dependent on the surgeons pre- and intraoperative interpretation. Although every patient was preoperatively assessed by CT scan and meticulous 2D planning was performed (using x-rays and CT scans) we did not use a dedicated 3D planning software. Furthermore, we decided to included different entities (Walch type B versus type C) and by that also different degrees of bony deficiency (type B2 versus B3 and C). This is also reflected in the fact, that only 28% of the cases needed intraoperative bone grafting. This, however, is to some degree also related to our preferred surgical technique using a lateralizing onlay humeral stem and normalized (i.e. not strongly lateralized) glenosphere position.

Although we found overall almost similar complication and revision rates between the groups and a subgroup analysis between the different Walch types in the study group did not show any significant differences, especially glenoid loosening/ pull out seems to occur slightly more frequent in patients with a glenoid deficiency (5 vs. 2). Four of the five patients with glenoid failures showed indeed a severe bony deficiency (3 patients with Walch type B3, one Walch type C). In 3 of them, intraoperative bony augmentation with a long peg glenoid baseplate was performed. Taking this into account the failure rate of bony augmented long peg glenoid reconstruction was 9% (3 out of 35). This is in accordance with the findings of Wagner et al. [31], who reported a large series of 40 patients undergoing revision RTSA with glenoid bone grafting, with 77% of implant survival at 5 years. The authors noted also concern when a lateralized glenoid component was implanted, although this effect has not shown any significance.

## Conclusion

RTSA seems to be a valuable treatment option for patients with primary (dysplasia) or secondary (wear) posterior glenoid deficiency. Although severe glenoid bone loss seems to be a risk factor for glenoid component failure, the overall complication and revision rate as well as clinical and radiographic outcome are comparable to RTSA in patients without compromised glenoid bone stock at a mean of 43 months.
